# Effects of Non-intubated Video-Assisted Thoracic Surgery on Patients With Pulmonary Dysfunction

**DOI:** 10.3389/fsurg.2021.792709

**Published:** 2022-01-06

**Authors:** Shiyu Deng, Yanyi Cen, Long Jiang, Lan Lan

**Affiliations:** ^1^Department of Anesthesiology, The First Affiliated Hospital of Guangzhou Medical University, Guangzhou, China; ^2^Department of Cardiothoracic Surgery, The First Affiliated Hospital of Guangzhou Medical University, Guangzhou, China; ^3^Guangzhou Institute of Respiratory Disease and China State Key Laboratory of Respiratory Disease, Guangzhou, China

**Keywords:** NIVATS, IVATS, pulmonary dysfunction, spontaneous respiration, PCP

## Abstract

**Background:** Non-intubated video-assisted thoracic surgery (NIVATS) can be safely performed in lung volume reduction surgery for patients with severe pulmonary dysfunction. However, there is still no cohort observation on the effects of NIVATS on patients with pulmonary dysfunction undergoing different types of thoracic procedures. This retrospective study aimed to observe the effects of NIVATS for this kind of patients.

**Methods:** Three hundred and twenty-eight patients with moderate to severe obstructive pulmonary dysfunction, who underwent video-assisted thoracic surgery (VATS), were retrospectively collected from June 1st, 2017 to September 30th, 2019. Patients in NIVATS were case-matched with those in intubated video-assisted thoracic surgery (IVATS) by a propensity score-matched analysis. The primary outcome was the comparison of perioperative values, the secondary outcome was the risk factors for postoperative clinical complications (PCP) which were identified by binary logistic regression analysis.

**Results:** After being matched, there were no differences in demographics and preoperative values of pulmonary function between NIVATS and IVATS groups. The duration of surgery and anesthesia had no difference (*P* = 0.091 and *P* = 0.467). As for the postoperative recovery, except for the mean intensive care unit (ICU) stay was longer in the IVATS group than in the NIVATS group (*P* = 0.015), the chest tube removal time and the postoperative hospital stay had no difference (*P* = 0.394 and *P* = 0.453), and the incidence of PCP also had no difference (*P* = 0.121). The binary logistic regression analysis revealed that the history of pulmonary disease, anesthesia method, and surgical location were risk factors of PCP.

**Conclusion:** For patients with pulmonary dysfunction when undergoing different types of thoracic procedures, the NIVATS can be performed as effectively and safely as the IVATS, and can reduce the ICU stay.

## Introduction

Lung volume reduction surgery (LVRS) has demonstrated significant improvements in respiratory function for patients with pulmonary dysfunction (1–3). However, LVRS may cause several complications, respectively leading to 5 and 59% mortality and morbidity rates (4).

Patients with pulmonary dysfunction usually underwent thoracic surgery with endotracheal intubation and general anesthesia, which can ensure intraoperative oxygenation and benefit airway management. However, patients need one-lung ventilation during operation, which may lead to pneumothorax on the ventilated side due to the fragile and inelastic lung tissue. In addition, many patients need postoperative ventilator support because of poor respiratory function compensation (5), and some patients are difficult to wean off the ventilator, resulting in lung infection, which increases complications and mortality (6).

A novel approach to this problem involves the increasing appliance of non-intubated video-assisted thoracic surgery (NIVATS). Perioperative morbidity may be reduced by avoiding positive pressure ventilation and employing a spontaneous respiration method (7), particularly in patients with poor cardiorespiratory performance (8). Small cases consisted of most early experience, which mainly dealt with patients with chronic respiratory failure or other comorbidities performed under epidural anesthesia (9).

However, a definitive conclusion on the efficacy of NIVATS on patients with pulmonary dysfunction cannot be drawn out on the small sample size (10). Besides, it was a lack of homology of the preoperative demographic (10, 11). Furthermore, many studies have just pointed out that NIVATS is feasible in patients with severe pulmonary dysfunction when undergoing LVRS, but there is no evidence for patients with moderate to severe pulmonary dysfunction when undergoing other types of thoracic procedures. Finally, there is scarce literature research on risk factors for postoperative clinical complications (PCP) after VATS for patients with pulmonary dysfunction. Identification of risk factors for PCP after VATS on a large cohort of patients is warranted. Therefore, the major outcome of this study is to make a retrospective comparison of NIVATS in patients with moderate to severe pulmonary dysfunction with similar patients of IVATS to undergo different types of thoracic procedures, and the secondary outcome was identified the risk factors for PCP for this kind of patients.

## Methods

**Figure 1:**
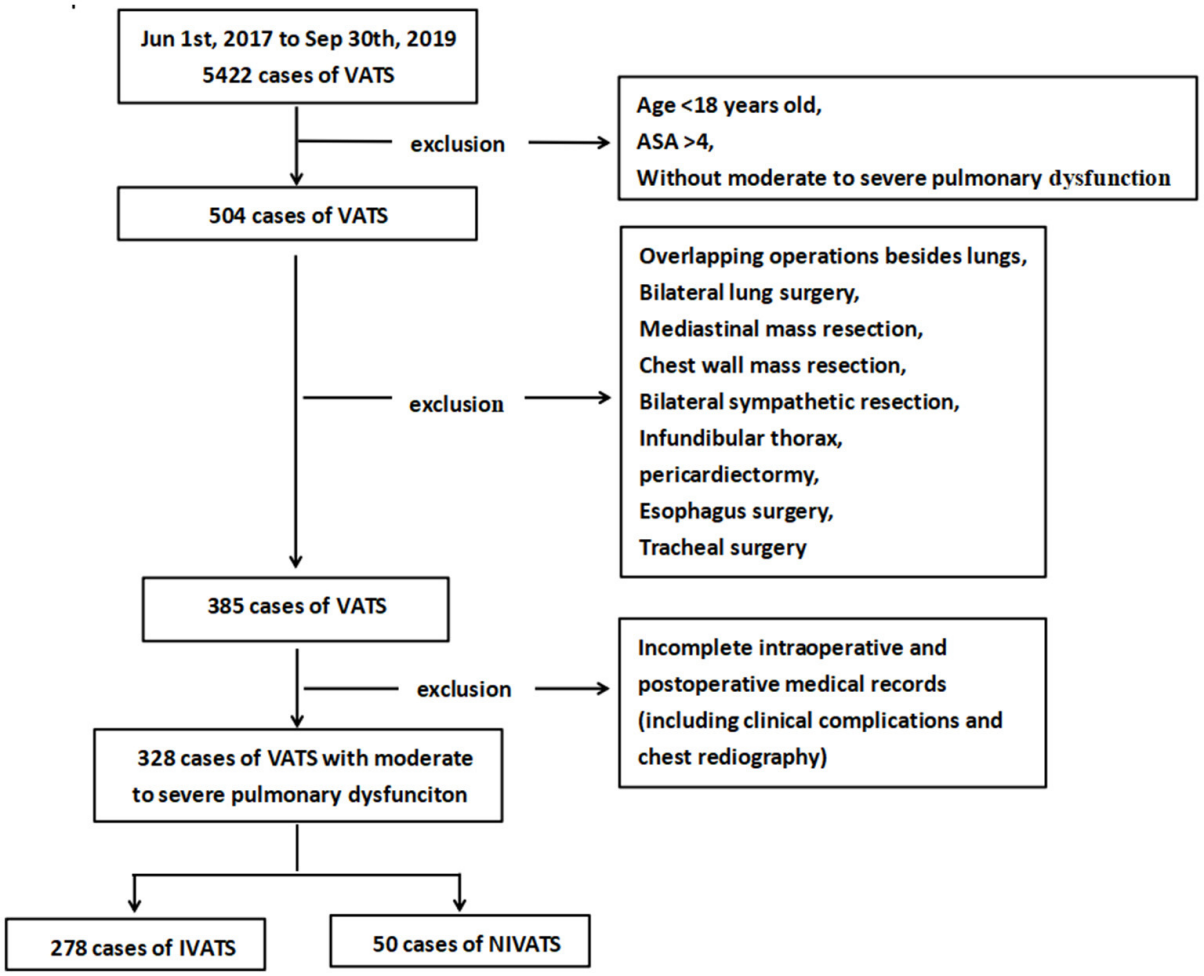
Flow chart of data collection. VATS, video-assisted thoracic surgery; NIVATS, non-intubated video-assisted thoracic surgery; IVATS, intubated video-assisted thoracic surgery.

This was a single-center retrospective cohort study. The perioperative clinical and radiological data have been stored according to a standardized protocol from June 1st, 2017 to September 30th, 2019. It was reviewed and approved by the Research Ethics Committee of the First Affiliated Hospital of Guangzhou Medical University, Guangzhou, China on February 9th, 2021, with the exemption from human subjects review and requirement of consent. Results of the NIVATS group were retrospectively compared with those of intubated video-assisted thoracic surgery group (IVATS group). Eligibility criteria were the same for both groups. The inclusion criteria were as follows: (1) adult patients over 18 years old underwent video-assisted thoracic surgery (VATS); (2) Patients with moderate-to-severe obstructive pulmonary dysfunction. According to the American Thoracic Society guideline, the preoperative forced expiratory volume in 1 second (FEV_1_) <60% predicted is defined as moderate to severe impairment of pulmonary function (12); (3) American Society of Anesthesiologists (ASA) score ≤ 3. Patients were excluded if they had a history of thoracic surgery. Patients were also excluded if they underwent overlapping operations besides lungs, bilateral lung surgery, mediastinal mass, thoracotomy, chest wall mass, bilateral sympathetic resection, infundibular thorax, pericardiectomy, pleural pathology, tracheal and esophageal surgery, or had an intraoperative conversion to endotracheal intubation due to bleeding, pleural adhesions, or other non-hypoxia factors. Patients with a lack of intraoperative values or incomplete postoperative medical records were also excluded (Figure 1).

### Anesthesia and Surgical Procedure

All patients received propofol target-controlled infusion (2–4 μg/ml), remifentanil (0.03–0.1 μg/kg/min), dexmedetomidine (0.5–1 μg/kg/h) for sedation and analgesic.

### IVATS Anesthesia

In the IVATS group, a Mallinckrodt double-lumen endobronchial tube was initiated under the assist of cisatracurium, and one-lung ventilation was commenced. A protective ventilation strategy was carried out to assure adequate oxygenation by low tidal volume 5–6 ml/kg and maximized exhalation time.

### NIVATS Anesthesia

The technique for NIVATS anesthesia has been already described in detail (13). Briefly, the laryngeal mask airway (LMA) was inserted into the pharynx and combined with regional anesthesia with intercostal and vagus block. Spontaneous ventilation was maintained during the operation. Fraction of inspired O_2_ (FiO_2_) was increased to maintain SpO_2_ ≥90% (14), and the mean arterial pressure (MAP) >60 mmHg was maintained by Dopamine or Norepinephrine. Permissive hypercapnia was acceptable, and pH <7.2 was set up as a safety limit for correction of hypercapnia (11).

### Surgical Procedure

The thoracoscopic procedures were similar in NIVATS and IVATS groups, which followed the consensus guidelines of the American Association for Thoracic Surgery (AATS) (15). The patient was placed in a full lateral decubitus position. The surgical procedure for each patient was determined according to the stage and location of the lesion in computed tomography images. Anatomical resection includes radical resection of lung cancer and segmental resection; Non-anatomical resection includes lung wedge resection, bullae resection, and lung volume reduction surgery. All patients were transferred to the post-anesthesia care unit (PACU) and then was sent back to the ward or intensive care unit (ICU). The blood cells analysis and blood gas analysis were performed on the first day after the operation. The PCP was based on the Clavien-Dindo classification. No PCP and fever were classified in Grade I; Dyspnea and arrhythmia were classified in Grade II; Thoracentesis and air leakage required replacing chest-tube were classified in Grade III; Mechanical ventilation was classified in Grade IV. The postoperative chest radiography results on the 1st and 4th days were compared by radiologists. Atelectasis, pulmonary exudation, or pleural effusion, were regarded as abnormal results. The chest tube was removed when serous fluid loss <200 ml in 24 h and no air leak following 2 h of tube clamping. Criteria for discharge were stable clinical conditions, oxygen saturation of 90% or above at rest, and all chest tubes removed. Postoperative hospital stay was defined as the number of hospitalized days after surgery.

### Statistical Analyses

All statistical analyses were performed using SPSS software (version 26.0, Chicago, IL, USA). The missing data (<5% of total values) were replaced by series mean. The selection bias of the NIVATS and IVATS groups was minimized by a propensity score matching analysis. Caliper matching method of 1:1 without replacement was used with caliper set as 0.2 standard deviations of the propensity scores. Balance examination was conducted by using standardized differences. The propensity score model development was done by including age, BMI, gender, and surgery type in the logistic regression model to predict the IVATS group. For continuous variables, restricted cubic spline function with three knots was also included, and non-significant cubic splines were excluded. We also examined potential interactions between predictor variables by stepwise logistic regression (entry significance = 0.2 and stay significance = 0.05). Owing to a large number of potential interactions, only two-way interaction was considered. The final model was then used to estimate the propensity scores.

Continuous data were presented as the mean± standard deviations for normal distribution, or as median (lower, upper quartiles) for skewness distribution. Dichotomous data were presented as numbers (%). The independent samples *t*-test was analyzed for continuous data between two groups. Mann-Whitney U-test was used for dichotomous data and skewed distributed data. The preoperative and postoperative counts of leukocyte and neutrophil were analyzed by paired *t*-test. The comparisons of clinical complications between two groups were analyzed by a Binomial test. *P* < 0.05 was considered to be statistically significant.

Binary logistics regression was used to analyze the linear regression relationship between preoperative factors and PCP to find risk factors. The Chi-square test or independent sample *t*-test was used to compare the perioperative clinical data between the two groups. *P* < 0.05 was indicated statistically significant.

## Results

A total of 328 patients with moderate to severe pulmonary dysfunction who underwent VATS were included, 278 patients in the IVATS group and 50 patients in the NIVATS group. Thirty-seven cases were identified in each group by matching propensity scores (Table 1).

**Table 1:** Demographic and clinical characteristics.

**Variable**	**Before matching**			**After matching**		
	**IVATS group**	**NIVATS group**	***P*-values**	**IVATS group**	**NIVATS group**	***P*-values**
	**(*n* = 278)**	**(*n* = 50)**		**(*n* = 37)**	**(*n* = 37)**	
Median age (years)	62.88 ± 11.35	55.94 ± 13.49	0.000	58.89 ± 13.55	60.41 ± 11.39	0.605
Gender (*n*, %)			0.080			1.000
Male	225 (81%)	35 (70%)		29 (78%)	29 (78%)	
Female	53 (19%)	15 (30%)		8 (22%)	8 (22%)	
Body mass index (kg/m^2^)	22.19 ± 3.48	22.11 ± 3.76	0.889	22.22 ± 3.53	21.84 ± 3.40	0.643
Comorbidity (*n*, %)						
Hypertension	83 (30%)	8 (16%)	0.044	8 (22%)	8 (22%)	1.000
Diabetes	32 (12%)	5 (10%)	0.756	6 (16%)	4 (11%)	0.499
Neurological disease	2 (1%)	2 (4%)	0.052	0	2 (5%)	0.154
Pulmonary disease	94 (34%)	11 (22%)	0.100	7 (19%)	11 (30%)	0.282
Smoking	138 (50%)	20 (40%)	0.210	21 (57%)	16 (43%)	0.248
Operation history	28 (10%)	7 (14%)	0.408	3 (8%)	5 (14%)	0.457
Hepatic renal insufficiency	5 (2%)	0	0.340	0	0	1.000
Revised cardiac risk index (*n*, %)			0.997			0.646
1 point	258 (93%)	47 (94%)		34 (92%)	35 (95%)	
2 points	20 (7%)	3 (6%)		3 (8%)	2 (5%)	
ThRCRI (*n*, %)			0.056			0.774
0	198 (71%)	42 (84%)		30 (81%)	29 (78%)	
1.5 point	75 (27%)	8 (16%)		7 (19%)	8 (22%)	
2 points	3 (1%)	0		0	0	
3 points	2 (1%)	0		0	0	
Types of thoracic procedure (*n*, %)			<0.001			0.166
Anatomical resection	194 (70%)	15 (30%)		21 (57%)	15 (41%)	
Non-anatomical resection	84 (30%)	35 (70%)		16 (43%)	22 (59%)	
Surgical location in the lung (*n*, %)			0.548			0.816
Upper lobe	152 (55%)	34 (68%)		23 (62%)	23 (62%)	
Middle lobe	19 (7%)	2 (4%)		3 (8%)	2 (5%)	
Lower lobe	81 (29%)	10 (20%)		10 (27%)	9 (24%)	
Whole lung	10 (3%)	1 (2%)		1 (3%)	1 (3%)	
Two lobes	16 (6%)	3 (6%)		0	2 (6%)	
Surgical site (*n*, %)			0.193			0.817
Left side	117 (42%)	26 (52%)		19 (51%)	18 (49%)	
Right side	161 (58%)	24 (48%)		18 (49%)	19 (51%)	
Pulmonary function tests (%)						
FVC% predicted	73.21 ± 14.38	62.73 ± 16.75	<0.001	68.33 ± 15.44	66.95 ± 17.12	0.717
FEV_1_% predicted	49.34 ± 10.14	47.88 ± 12.46	0.478	47.21 ± 8.84	47.94 ± 13.10	0.780

*Continuous data were presented as the mean ± sd. Dichotomous data were presented as numbers (%). ThRCRI, the Thoracic revised cardiac risk index; FVC% predicted, the forced vital capacity in percent of predicted; Fev_1_% predicted, the forced expiratory volume in the first second in percent of predicted*.

### Comparison of Preoperative Values

There were no statistically significant differences in age, gender, BMI, comorbidity, ThRCRI, types of thoracic procedure, surgical location, surgical site, and the values of pulmonary function test between IVATS and NIVATS groups after matching (*P* > 0.05) (Table 1).

### Comparison of Intraoperative and Postoperative Values

The duration of surgery and anesthesia had no difference (*P* = 0.091 and *P* = 0.467). As for the postoperative recovery, the duration of chest-tube indwelling and the postoperative hospital stay had no difference (*P* = 0.394 and *P* = 0.453), except for the mean ICU stay was longer in the IVATS group than in the NIVATS group (*P* = 0.015). The PCP based on the Clavien-Dindo classification also had no difference (*P* = 0.121) (Table 2).

**Table 2:** Comparison of intraoperative and postoperative variables.

**Variables**	**Before matching**			**After matching**		
	**IVATS group**	**NIVATS group**	***P-*values**	**IVATS group**	**NIVATS group**	***P-*values**
	**(*n* = 278)**	**(*n* = 50)**		**(*n* = 37)**	**(*n* = 37)**	
Surgical duration (min)	140 (95–190)	58 (40–120)	<0.001	115 (75–190)	75 (50–140)	0.091
Anesthesia duration (min)	220 (180–270)	135 (118–230)	<0.001	210 (150–272)	170 (120–263)	0.467
Mean ICU stay (days)	1.00 (0.00–1.00)	0.00 (0.00–0.00)	<0.001	1 (0–1)	0 (0–0)	0.015
Chest-tube duration (days)	3.00 (2.00–5.00)	3.00 (0.00–5.00)	0.008	3 (3–5)	3 (1–6)	0.394
Postoperative hospital stay (days)	6.00 (4.00–7.00)	7.00 (4.00–9.00)	0.187	6 (4–9)	7 (5–10)	0.453
Postoperative clinical complications based on Clavien-Dindo classification (*n*, %)			0.078			0.121
Grade I	224 (81%)	34 (68%)		31 (84%)	24 (65%)	
Grade II	31 (11%)	13 (26%)		2(5%)	11 (30%)	
Grade III	5 (2%)	0		0	0	
Grade IV	18 (6%)	3 (6%)		4 (11%)	2 (5%)	

*Continuous data were presented as median (lower, upper quartiles). Dichotomous data were presented as numbers (%). ICU, intensive care unit*.

### Risk Factors of Postoperative Clinical Complications

Logistic regression analysis showed that pulmonary disease, anesthesia method, surgery location in the upper lobe, were the risk factors for PCP (*P* < 0.05) (Table 3).

**Table 3:** Risk factors of postoperative clinical complications.

**Variables**	***P*-value**	**OR (95% CI)**
Pulmonary disease	0.001	3.033 (1.549–5.938)
Anesthesia method	0.015	2.932 (1.228–7.000)
Surgery location	0.009	
Upper lobe	0.019	4.271 (1.271–14.346)
Middle lobe	0.041	0.429 (0.191–0.965)

*The anesthesia method referred to endotracheal intubation with double-lumen tube (IVATS group) or spontaneous breathing with laryngeal mask airway (NIVATS group). CI, confidence interval; OR, odds ratio*.

### The Comparison of Blood Cells and Blood Gas Analysis

**Figure 2:**
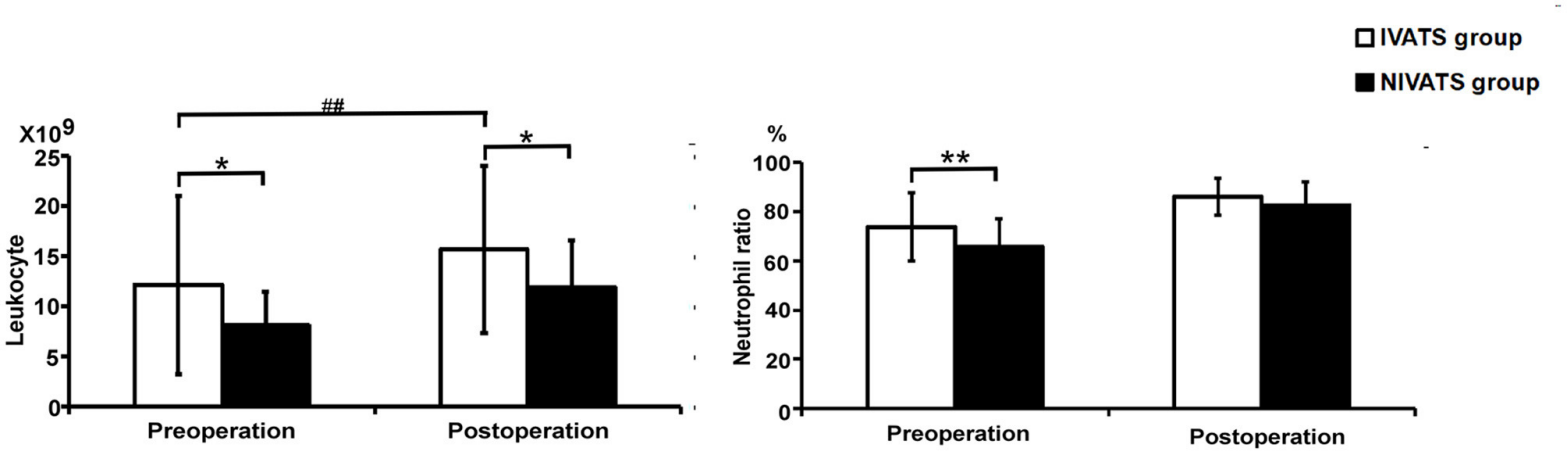
Blood cells analysis (the left is leukocyte and the right is neutrophil ratio) in the IVATS group and NIVATS group before and after the operation. *P-value presents the comparison of values between IVATS and NIVATS group (*presented *P* < 0.05 and **presented *P* < 0.01) ^#^*P*-value presents the comparison of preoperative and postoperative values in the IVATS group (^#^presented *P* < 0.05 and ^##^presented *P* < 0.01).

Preoperative and postoperative counts of leukocytes were higher in the IVATS group than those in the NIVATS group (*P* = 0.011 and *P* = 0.021), and the postoperative counts of leukocytes were higher than preoperative ones in the IVATS group (*P* < 0.001). The preoperative values of neutrophils were higher in the IVATS group (*P* = 0.007), but the values of preoperative and postoperative nurtrophils had no difference between the IVATS and NIVATS groups (*P* = 0.465 and *P* = 0.492) (Figure 2).

**Figure 3:**
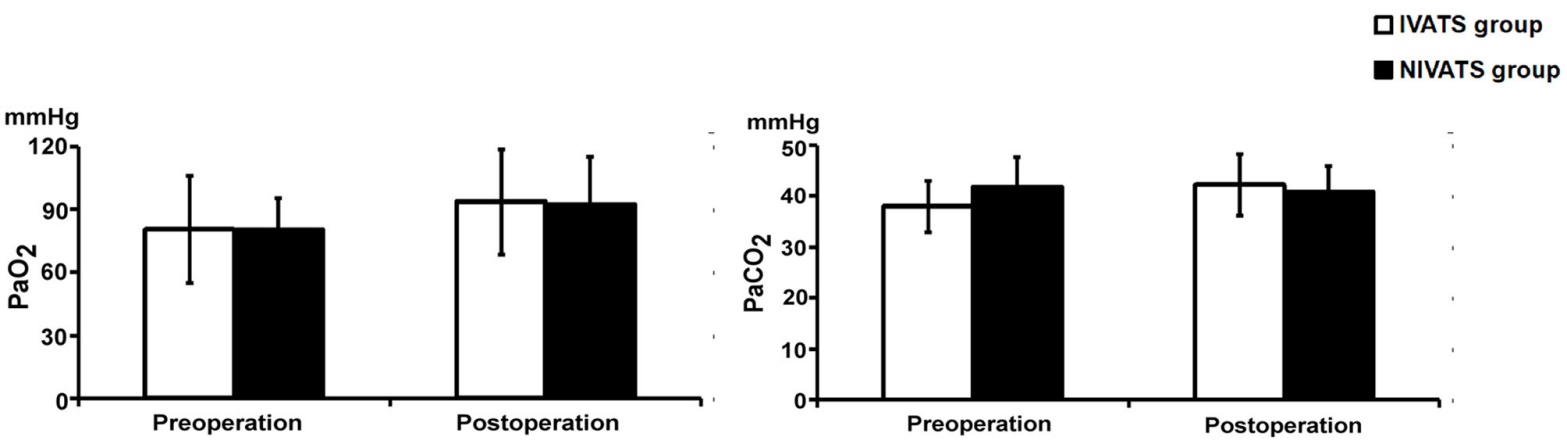
Blood gas values changed in the IVATS group and NIVATS group. Before and after operation, the PaO_2_ (Left) and PaCO_2_ (Right) of the two groups were compared. No significant difference of preoperative and postoperative values between the two groups.

No significant difference in preoperative and postoperative values of PaO_2_ and PaCO_2_ between the two groups (*P* > 0.05) (Figure 3).

## Discussion

The major finding of this retrospective study revealed that no patients of the NIVATS group were reintubated due to hypoxia. It was believed that, for patients with moderate to severe pulmonary dysfunction, the methods of NIVATS, did not affect the duration of surgical and anesthesia, and did not affect the incidence of PCP, when compared with IVATS, but the NIVATS decreased the length of ICU stay. Postoperative clinical complications occurred in 71 (21.6%) of 328 patients. The main risk factors for PCP were the pulmonary disease, anesthesia method, surgery location in the upper lobe.

With the improvement of surgical techniques and anesthesia methods, patients with pulmonary dysfunction, which was taboo in surgery decades ago, can also receive thoracic surgery. This population mostly is older, with multiple comorbidities, and poor pulmonary function, which makes the perioperative period full of challenges. Video-assisted thoracic surgery is becoming a standard of care (16), and can reduce the incidence of respiratory complications in patients with poor pulmonary function (17). At present, the NIVATS also has been performed in this kind of patients with good outcomes (10, 11).

Our study confirmed that patients with moderate to severe pulmonary dysfunction can tolerate different types of thoracic procedures when initiated NIVATS. Complex and time-consuming anatomical procedures were more than 40% in NIVATS groups. The duration of anesthesia and surgery were the same as those in IVATS. As a result, the ICU stay was shorter than that of the IVATS group. Finally, the postoperative hospital stay and chest-tube duration, and the incidence of PCP were improved, and these improvements did not differ from those achieved in the IVATS group. When VATS was performed for patients with pulmonary dysfunction, endotracheal intubation general anesthesia was initiated in the past. Indeed, the positive pressure ventilation may be harmful, when in contrast to spontaneous respiration which tidal volume is created by negative inspiratory pressure. Positive pressure ventilation can aggravate fragile lungs by irreversible barotrauma (18). Non-intubated thoracic surgery aims to do as least damage as possible.

Contrary to the intubated method, NIVATS offers an option to avoid more damage on pathological lungs by preserving the negative inspiratory pressure instead of positive inspiratory pressure and maybe currently the best option for fragile patients. Furthermore, general anesthesia applied on patients with pulmonary dysfunction would have carried the risk of it being difficult to wean off these patients from ventilators (5, 6). Therefore, NIVATS without muscle relaxants and maintaining spontaneous ventilation may avoid this concern. Non-intubated video-assisted thoracoscopy resulted in a significantly shorter ICU stay, which offered the double advantage of less risk of iatrogenic infection, reducing medical costs because of quicker discharge, makes more space on the patient's waiting list and also saves human resources because ICU staff can deal with patients staying less time in the intermediate care or the ICU. More and more samples have confirmed that fragile patients can benefit from NIVATS with good outcomes from thoracic surgery (19, 20).

Spontaneous respiration inevitably leads to hypercapnia resulting from intraoperative CO_2_ accumulation. In this case, the benefit of NIVATS outweighs the risk of intraoperative hypercapnia due to its fast recovery, and on the other hand, these patients have poor pulmonary function, the basic level of CO_2_ is high, so they can tolerate high levels of hypercapnia. Some of the patients included in this study were ASA grade 3, which implied that NIVATS could also be extended to patients higher than ASA II. But this requires that, firstly, Anesthesiologists need to have much experience of NIVATS, for the reason that it may have to convert to endotracheal intubation due to unpredictable conditions, such as bleeding or pleural adhesion (21); secondly, the surgeon should have rich experience in thoracoscopy, preferably more than 5 years, and can complete various types of thoracoscopic procedures (22); thirdly, the operation time should not be too long and the surgical procedure is not too difficult. Since long-term spontaneous breathing during NIVATS may lead to hypoxia and hypercapnia. Previous unilateral lobectomy and contralateral wedge resection (23), as well as lung volume reduction surgery in patients with COPD (7, 24–26), these patients with severe pulmonary dysfunction, are more suitable for NIVATS.

Licker showed that an FEV_1_ <60% was associated with an increase in respiratory complications (27). Awake non-resectional LVRS has been proved feasible and safe for patients with severe pulmonary dysfunction with a faster recovery (11, 28, 29) and satisfactory 6-month outcome (23), and with less prolonged air leak (30). As for patients with moderate pulmonary dysfunction, NIVATS can also be proved to work (10). Our anesthesia method is similar to Wang's (10), but Wang's sample size is small and lack of the control group. The current analysis results add to the previous findings that, as for patients with moderate to severe pulmonary dysfunction, the NIVATS can offer similar clinical outcomes with IVATS when performed different types of thoracic procedures, especially with a reduced duration of ICU stay.

The anesthesia method was one of the main risk factors for PCP, which can be preluded that the complications of intubated general anesthesia, such as residual neuromuscular blockade, ventilator-induced lung injury, and airway trauma, can be avoided in NIVATS (31–34). Furthermore, refraining from general anesthesia in patients combined with pulmonary diseases, such as COPD, is associated with lower incidences of pulmonary infection, long-term ventilator dependence, and postoperative reintubation (35). Surgery location in the upper lobe was also a risk factor for PCP. The possible reason was that, under the effect of intrathoracic pressure, the alveolar expansion of the lower lobe is inferior to that of the upper lobe. When the upper lobe was removed, the blood flow of the lower lobe increased, but the ventilation did not improve immediately after the operation, which easily caused the imbalance of ventilation to perfusion, thus increased the complications such as postoperative hypoxia. In addition, upper lobe surgery accounted for more than 60% of all operations in this study. Accordingly, the probability of postoperative complications was also increased.

The elements of fast-track surgery—regional analgesia, smaller incision, minimizing opiate use, rapid metabolism of anesthetics, and a goal-directed fluid therapy—are not novelties to thoracic surgery (36). And the NIVATS contains all these elements. Non-intubated video-assisted thoracic surgery can accelerate postoperative rehabilitation, and our results also suggested that NIVATS can provide postoperative clinical results similar to IVATS. But in our study, patients with higher preoperative values of leukocyte and neutrophil mostly initiated IVATS, indicating that patients with severe preoperative infection are still recommended to choose intubated general anesthesia, which is owing to its effectiveness draining sputum during operation, and avoiding intraoperative hypoxia.

## Limitations

Our study has several weaknesses. Firstly, it was a single-center study that is partly retrospective and the number of patients was limited. Secondly, the surgical procedures were variable, rather than a single procedure. But this can further show that NIVATS is feasible for different types of thoracic procedures. Thirdly, although previous literature has reported the effectiveness of NIVATS in patients with severe pulmonary dysfunction, the results of NIVATS in patients with moderate to severe pulmonary dysfunction is a useful supplement to previous studies. Fourth, patients, who had an intraoperative conversion to endotracheal intubation due to surgical factors, not non-hypoxia factors, were excluded from the study. A small number of patients may be excluded, but this allowed a more homogeneous sample. In addition, the effects of the two anesthesia methods on the postoperative recovery were mainly observed. Thus, only the successful completion of anesthesia can be included in the study. Patients excluded due to surgical factors would not affect the risk factor analysis.

## Conclusion

Our study results have shown that patients with moderate and severe obstructive ventilation dysfunction when undergoing different types of thoracoscopic surgery, NIVATS show similar outcomes with IVATS during postoperative hospitalization, except for the decreased the length of ICU stay. In other words, for patients with moderate to severe obstructive ventilation dysfunction, the NIVATS or IVATS does not affect the short-term postoperative rehabilitation, both NIVATS and IVATS are feasible and safe. In the future, we need more randomized controlled trials to confirm this conclusion, and we need to observe the comparison of long-term rehabilitation.

## Data Availability Statement

The original contributions presented in the study are included in the article, further inquiries can be directed to the corresponding author/s.

## Ethics Statement

The studies involving human participants were reviewed and approved by Medical Ethics Committee of the First Affiliated Hospital Guangzhou Medical University. The ethics committee waived the requirement of written informed consent for participation.

## Author Contributions

SYD collected the clinical data and writing the manuscript. LL participated in the drafting and critically revised the manuscript. YYC and LJ supervised the study and conducting statistical analyses. All authors substantially contributed to the manuscript.

## Conflict of Interest

The authors declare that the research was conducted in the absence of any commercial or financial relationships that could be construed as a potential conflict of interest.

## Publisher's Note

All claims expressed in this article are solely those of the authors and do not necessarily represent those of their affiliated organizations, or those of the publisher, the editors and the reviewers. Any product that may be evaluated in this article, or claim that may be made by its manufacturer, is not guaranteed or endorsed by the publisher.

## Abbreviations

VATS: video-assisted thoracic surgery
NIVATS: non-intubated video-assisted thoracic surgery
IVATS: intubated VATS
ICU: intensive care unit
LVRS: lung volume reduction surgery
PCP: postoperative clinical complications
FEV_1_: forced expiratory volume in 1 second
LMA: laryngeal mask airway
FiO_2_: fraction of inspired O_2_
MAP: mean arterial pressure
AATS: American Association for Thoracic Surgery
PACU: post-anesthesia care unit.
